# Effect of Irradiation on Cell Transcriptome and Proteome of Rat Submandibular Salivary Glands

**DOI:** 10.1371/journal.pone.0040636

**Published:** 2012-07-06

**Authors:** Raluca Stiubea-Cohen, Ran David, Yoav Neumann, Guy Krief, Omer Deutsch, Batia Zacks, Doron J. Aframian, Aaron Palmon

**Affiliations:** 1 Faculty of Dental Medicine, Institute of Dental Sciences, The Hebrew University of Jerusalem, Jerusalem, Israel; 2 Salivary Gland Clinic and Saliva Diagnostic Laboratory, Department of Oral Medicine, The Hebrew University of Jerusalem, Hadassah Medical Center, Jerusalem, Israel; St. Georges University of London, United Kingdom

## Abstract

Salivary glands (SGs) are irreversibly damaged by irradiation (IR) treatment in head and neck cancer patients. Here, we used an animal irradiation model to investigate and define the molecular mechanisms affecting SGs following IR, focusing on saliva proteome and global transcription profile of submandibular salivary gland (SSG) tissue.

We show that saliva secretion was gradually reduced to 50% of its initial level 12 weeks post-IR. Saliva protein composition was further examined by proteomic analysis following mass spectrometry (MS) analysis that revealed proteins with reduced expression originating from SSGs and proteins with increased expression derived from the serum, both indicating salivary tissue damage. To examine alterations in mRNA expression levels microarray analysis was performed. We found significant alterations in 95 genes, including cell-cycle arrest genes, SG functional genes and a DNA repair gene.

Tissue damage was seen by confocal immunofluorescence of α-amylase and c-Kit that showed an increase and decrease, respectively, in protein expression. This was coherent with real-time PCR results.

This data indicates that IR damages the SSG cells' ability to produce and secrete saliva and proteins, and maintain the physiological barrier between serum and saliva. The damage does not heal due to cell-cycle arrest, which prevents tissue regeneration. Taken together, our results reveal a new insight into IR pathobiology.

## Introduction

Each year, 500,000 patients are diagnosed with head and neck cancer worldwide, and most of them receive irradiation (IR) treatment [1]. This mode of treatment has a pronounced effect on the salivary glands (SGs), resulting in irreversible damage to the parenchymal tissue [2]. Irradiated patients suffer from diminished ability to secrete saliva, with consequent eating and vocal disturbances, as well as frequent mucosal infections, rampant dental caries, periodontal disease and episodes of considerable pain and coughing, all of which significantly decrease their quality of life [3]. In recent years, we and others have concentrated on methods to restore normal SG function in these patients [4], [5]. The notion of SG regeneration is based on the assumption that autologous SG graft cells can be isolated before initializing IR therapy, cultivated, and preserved during the IR period. Upon treatment completion, cells can then be implanted into the irradiated gland, replacing the functionally damaged ones. One pivotal point that still needs to be addressed toward this novel strategy is the detailed effect of IR on the salivary transcriptome and proteome, to facilitate our ability to monitor treatment outcome at the molecular level, and not only by measuring saliva output.

Several animal models have been established to examine SG radiosensitivity, a common one being the single 15-Gy dose rat model implemented in this study. This model has been used to describe time kinetics of saliva output, limited protein composition (mainly amylase as a marker of acinar function) and tissue structure responses following IR [2], [6], [7], [8], [9], [10], [11], [12], [13], [14], [15]. Those studies have demonstrated that in the post-IR stages, function is damaged, as reflected by a 50% loss of salivary flow, reduction in α-amylase activity and subsequent glandular shrinkage. This has been attributed to reduced acinar cell activity, and loss of acinar cells as a result of apoptosis and acinar stem cell death [2], [11], [12]. Unlike other radiosensitive tissues, SG cells proliferate slowly and are highly differentiated. Thus, the effect of IR cannot be attributed solely to rate of tissue proliferation.

To define the molecular mechanisms affecting SGs following IR, we studied the effect of IR on saliva output, global transcription profile of submandibular SG (SSG) tissue and whole saliva proteome before and up to 12 weeks after IR.

## Materials and Methods

### Animals

Female Sprague-Dawley rats (200–225 g) were used as the animal model. All experiments were approved by the Ethical Committee on animal testing of the Hebrew University (NIH approval number: OPRR-A01-5011, Research number MD-07-10472-4). All animals were treated according to procedures approved by the Animal Care and Use Committee at our institute and were monitored continuously for any signs of distress. Animals were kept at 22±2°C.

### Irradiation treatment

Salivary glands were locally irradiated with a single dose of 15Gy of X-rays delivered by Clinac DBX linear accelerator with 6 MV photon energy (Varian Medical Systems, Palo Alto, CA, USA). This dose is known to induce sufficient functional damage without compromising the general health of the animals. Rats were anesthetized with ketamine (100 mg/ml) and xylazine (20 mg/ml) at 100 µl/100 g body weight. Rats were placed on their backs facing up and the IR field was determined such that only the head and neck regions were exposed. Control animals were anesthetized and placed in a box but were not irradiated.

### Saliva collection and preparation

Whole saliva was collected from anesthetized animals before and after irradiation (4–9 animals per group) for 15 min after subcutaneous administration of pilocarpine (2 mg/kg body weight). Saliva samples were immediately put on ice and thereafter were centrifuged at 14,000 *g* for 20 min at 4°C to remove insoluble materials, cell debris and food remnants. Saliva quantity was determined gravimetrically, assuming a density of 1 g/ml. Whole saliva secretion was measured before and 4, 8 and 12 weeks post-IR. Saliva flow rate was normalized to body weight. Saliva control values were set at 100%, and saliva outputs of IR animals were presented as percentage of the mean control value. Protein concentration was determined using the Bio-Rad protein assay according to Bradford [16] (Bio-Rad, Hercules, CA, USA).

### 2-DE (two-dimensional gel electrophoresis) analysis

Saliva samples pooled from at least four animals per time point, repeated at least twice, were frozen at −80°C and lyophilized overnight. Sediments were dissolved in 7 M urea, 2 M thiourea and 4% (w/v) CHAPS and stored at −80°C until analysis.

For analytical gels, samples of 45 mg protein were rehydrated and subjected to isoelectrofocusing on 18-cm long pH 3–10 NL Immobiline DryStrips gels (Amersham Biosciences, Uppsala, Sweden) as we previously described [17].

### 2-DE Imaging

Gels were scanned using a computerized GS-800 calibrated densitometer (Bio-Rad) and protein spots were detected and quantified using Redfin software (Ludesi, Malmo, Sweden). Several limitations exist in 2D gel analysis as a result of gel-to-gel variation as well as variability in the staining method. To overcome these limitations, all gels were subjected to the same conditions simultaneously for the first and second dimensions. Normalization was performed by the total density in image method of Redfin to quantify spot intensities and minimize staining variation between gels.

### Mass spectrometry (MS) identification and database search

For MS identification, a 2-DE gel containing 500-µg protein samples was fixed in 50% (v/v) ethanol, 12% (v/v) acetic acid for 2 h. Proteins were visualized by staining for 16 h with Coomassie Brilliant Blue G250 (Fluka, Buchs, Switzerland), followed by destaining in 20% ethanol. Electrophoretically separated protein spots were selected by matching to those on a silver-stained 2-D gel and were excised under sterile conditions, as previously described [17]. The MS data were clustered and analyzed using Sequest software (version 3.31; J. Eng and J. Yates, University of Washington and Finnigan, San Jose, CA, USA) and Pep-Miner [18] searching against the rat part of the Uniprot database.

### RNA isolation

Before IR and 4, 8 and 12 weeks post-IR, rats were sacrificed and SSGs were removed for RNA isolation using the RNeasy Micro Kit (Qiagen, Hilden, Germany) according to the manufacturer's instructions. RNA concentration was measured using a Nanodrop ND-100 spectrophometer.

### Microarray

Total RNA (1 µg) from samples pooled from at least four animals per time point was amplified and labeled with a fluorescent dye (either Cy3 or Cy5) using the Low RNA Input Linear Amplification & Labeling kit (Agilent Technologies, Palo Alto, CA, USA) following the manufacturer's protocol. The amount and quality of the resulting labeled cRNA was measured in a Nanodrop ND-100 spectrophometer. Equal amounts of Cy3- or Cy5-labeled cRNA were hybridized to the 4×44 K Agilent Whole Rat Genome Oligo Microarray for 17 h at 60°C in an Agilent DNA-Microarray Hybridization Oven. The arrays were then washed using the Gene Expression Wash Buffer Kit from Agilent. The microarray was scanned using a GenePix4000B Scanner (Axon, Union City, CA, USA) and the data were extracted from the resulting images using GenePix Pro 4.1 software (Axon). The result files (gpr) produced by GenePix were analyzed utilizing the LIMMA [19] software package, available from CRAN site (http://www.r-project.org). Spots flagged as not found or absent in GenePix were removed by filtering. The Cy5 and Cy3 intensities within each array were normalized using the print-tip locally weighted scatter plot smoothing (LOESS) function, with no background correction applied. To identify differentially expressed genes, a parametric empirical Bayesian approach implemented in LIMMA was used [19]. A moderated *t* test was performed in parallel, with the use of a false discovery rate [20] correction for multiple testing. LIMMA calculated an emission intensity A value [A = (log_2_(Cy5)×Cy3)/2] where Cy5 and Cy3 are the normalized emission intensities of each feature. A confidence level of *P*<0.05 was used to pinpoint significantly differentiated genes. Genes had to have an A value (average expression level for the gene across all arrays and channels) of more than 8.5, thus excluding faint emissions. The differentially expressed genes for each binary comparison retrieved by LIMMA were clustered using EXPANDER software [21]. A functional analysis of KEGG (Kyoto Encyclopedia of Genes and Genomes) pathways and functional categories was performed for all genes using David online software [22].

### Real-time PCR

Total RNA from at least three animals per time point was reverse-transcribed using the High Capacity cDNA Reverse Transcription Kit (Applied BioSystems/Ambion, Austin, TX, USA) according to the manufacturer's instructions. cDNA was amplified using the 7300 real-time PCR system (Applied Biosystems) with TaqMan gene expression assay for α-amylase and cyclin D1 genes, *Cdh22*, *p21*, *p57*, *CysS*, *Psp*, *Prb1*, *Muc19*, *Mgmt, Ywhaq*, and c-KIT and GAPDH genes (Applied Biosystems). Data were analyzed with 7300 System SDS Relative Quantification Software version 1.4.0.25 (Applied BioSystems).

### Histolochemical staining, immunofluorescence and confocal microscopy

The histochemical and immunofluorescence assay was performed as follows. SSGs were excised and fixed in 4% (v/v) paraformaldehyde in PBS overnight at 4°C. The tissue was embedded in paraffin, and 5-µm sections were cut. The sections were de-paraffinized and rehydrated in a graded ethanol series. Cell morphology was visualized by routine histologic techniques using hematoxylin-eosin (H&E) staining. For immunofluorescence the sections were treated for antigen retrieval by boiling in citrate buffer (10 mM, pH 6.0, 0.05% v/v Tween 20) for 10 min at 100°C and cooled to room temperature for 30 min. Nonspecific antibody binding was blocked by 60 min incubation with 5% (v/v) goat serum and 0.1% (v/v) Triton X-100 in PBS at room temperature. The antibodies for amylase (2 µg/ml, mouse monoclonal IgG; Santa Cruz Biotechnology, Santa Cruz, CA, USA) and c-KIT (CD117, 2 µg/ml, Santa Cruz Biotechnology) were diluted 1∶50 and 1∶25, respectively, with 1% goat serum and 0.1% Triton X-100 in PBS, and incubated overnight at 4°C. Then the slides were incubated for 60 min at room temperature with secondary antibody Alexa Fluor 555-conjugated donkey anti-mouse IgG (Invitrogen, Carlsbad, CA, USA) or Alexa Fluor 647-conjugated goat anti-mouse IgG (Invitrogen, Carlsbad, CA, USA) at a dilution of 1∶500. For nuclear staining, 4,6-diamidino-2-phenylindole (DAPI; 1 µg/ml; KPL, Gaithersburg, MD, USA) was added to samples and incubated for 5 to 7 min at room temperature. Slides were then rinsed and cover-slipped with fluorescent mounting medium (DAKO, Carpinteria, CA, USA). The stained preparations were evaluated using a confocal laser scanning microscope (Model 710, Zeiss).

### SGIE isolation and colony forming efficiency

SSG integrin α6β1-positive cells (SGIE) were immuno-magnetic isolated as we previously described [23], [24]. To test their colony forming efficiency, 5000 cells were seeded on irradiated NIH 3T3 fibroblasts for 10 days on a 35-mm Petri dish. Then colonies were fixed with 4% formaldehyde (Sigma) for 10 min at room temperature. After counter-staining with 1% Rhodamine B solution (Sigma), colonies were manually counted under light microscopy

### Statistical analysis

Statistical analysis of MS data was performed as described in above. Statistical analysis of microarray data was performed as described in section above. Saliva flow rate, real-time PCR validation and assessment of colony forming efficiency were performed by One-way ANOVA, followed by Tukey test. Results are presented as mean values ± SD. *P*<0.05 was considered statistically significant.

## Results

### Effect of IR on saliva secretion and proteome

The most fundamental function of the SGs is to produce and secrete saliva. We therefore studied the effect of 15-Gy head and neck IR on saliva output.

**Figure 1:**
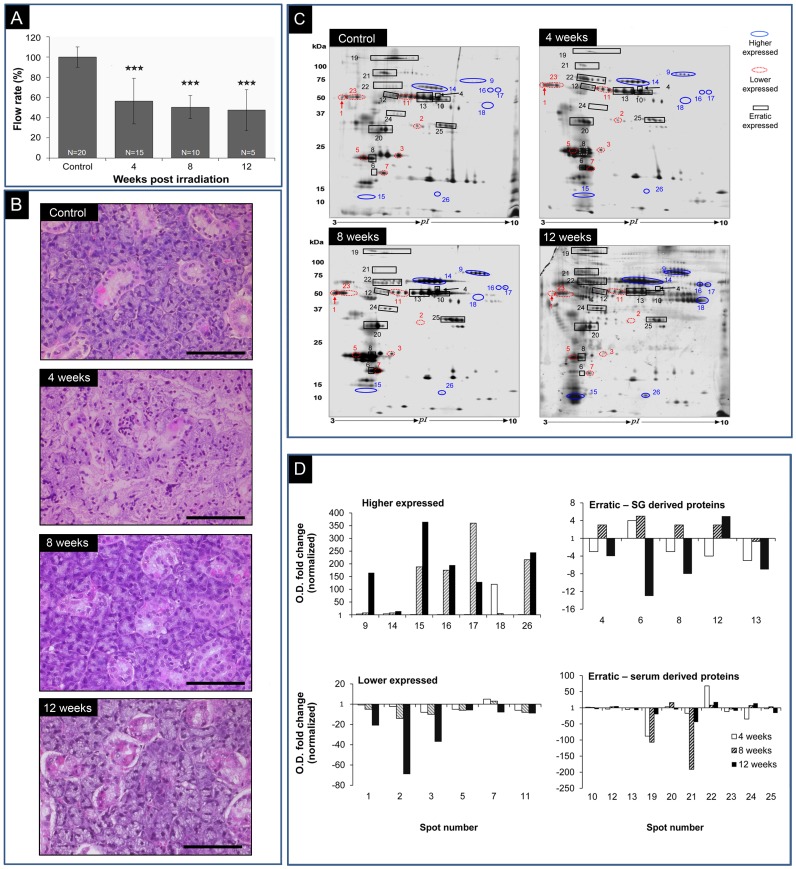
Effect of 15-Gy head and neck irradiation on saliva secretion. A. Saliva flow rate after irradiation: normalized, pilocarpine-induced saliva secretion from non-irradiated (control) and 4-, 8- and 12-week post-irradiation rats. The number of rats used in each experiment is indicated inside the bars. ***P<0.001 vs. control. B. H&E staining of rat submandibular gland tissue from control and irradiated rats. C. Representative 2-D electrophoretic analysis of pooled saliva before and after 15-Gy irradiation: saliva protein (45 µg) from non-treated animals, and animals 4 weeks, 8 weeks and 12 weeks post-irradiation were separated by 2-D electrophoresis. Proteins with reduced expression during the 12 weeks following IR are represented by red ellipses, those with higher expression by blue ellipses and those with erratic expression by black rectangles. D. Changes in normalized optical density ratios and MS identification of selected cluster spots after 15-Gy irradiation: identified spot proteins are shown in decreasing order of matched peptides.

Fig. 1A demonstrates the effect of IR on saliva secretion before IR and for 12 weeks post-IR. Four weeks post-IR, saliva decreased to 56% of the output in control animals, continued to decrease to 50% at 8 weeks, and then remained at that level until at least 12 weeks post-IR. Fig. 1B shows H&E staining of submandibular salivary glands before and 4, 8 and 12 weeks after IR. Four weeks after IR structural changes were found in parts of the gland, including aberration in acini organization and reduction in the numbers of ducts. Eight and 12 weeks after IR gland structure appeared normal without signs of fibrosis.

Saliva protein composition was determined by proteomic analysis before and 4, 8 and 12 weeks after IR (Fig. 1C), followed by MS identification (Table S1). Among the protein spots with altered intensities, as determined by 2-DE after IR, three protein groups were detected: those showing increased expression (Fig. 1C, blue ellipses), those showing reduced expression (Fig. 1C, red ellipses), and those with erratic expression (Fig. 1C, black rectangles).

MS identification of the up-regulated proteins showed that most of them were derived from the serum, including serum albumin precursor, serotransferrin, immunoglobulins and complement components (Table S1, spots 9, 14–18, and 26, Fig. 1D). MS identification of the down-regulated proteins (Table S1, spots 1–3, 5, 7 and 11, Fig. 1D) showed that most of them originated from the SG, including α-amylase, parotid secretory protein, prolactin-induced protein and von Ebner gland protein 1 and 2.

The third group, containing proteins with erratic expression, included proteins derived from both the SG (parotid secretory protein, α-amylase, prolactin-induced protein, von Ebner gland protein 1 and 2, and cysteine-rich secretory protein 1 precursor) and serum (serum albumin precursor, serotransferrin, apolipoprotein E precursor, immunoglobulins and complement components) (Table S1, Fig. 1D). While the serum-derived proteins tended to return to normal levels 12 weeks post-IR, most of the SG-derived proteins, except for spot 12, tended to be at reduced levels 12 weeks post-IR (Table S1).

### Effect of IR on SSG transcriptome and SGIE colony forming efficiency

We identified 95 genes that were significantly differentially expressed as a result of IR using microarray (Table S2). We used these differentially expressed genes for further functional annotation of pathways and functional categories using the David online database (http://david.abcc.ncifcrf.gov/). A significant over-representation of genes in the following functional categories was observed: endoplasmic reticulum, signal, emp24/gp25L/p24, enzyme inhibitor activity, protein transport, cell redox homeostasis, nucleotide binding, golgi apparatus, proteolysis, protein folding and secretory granules.

**Figure 2:**
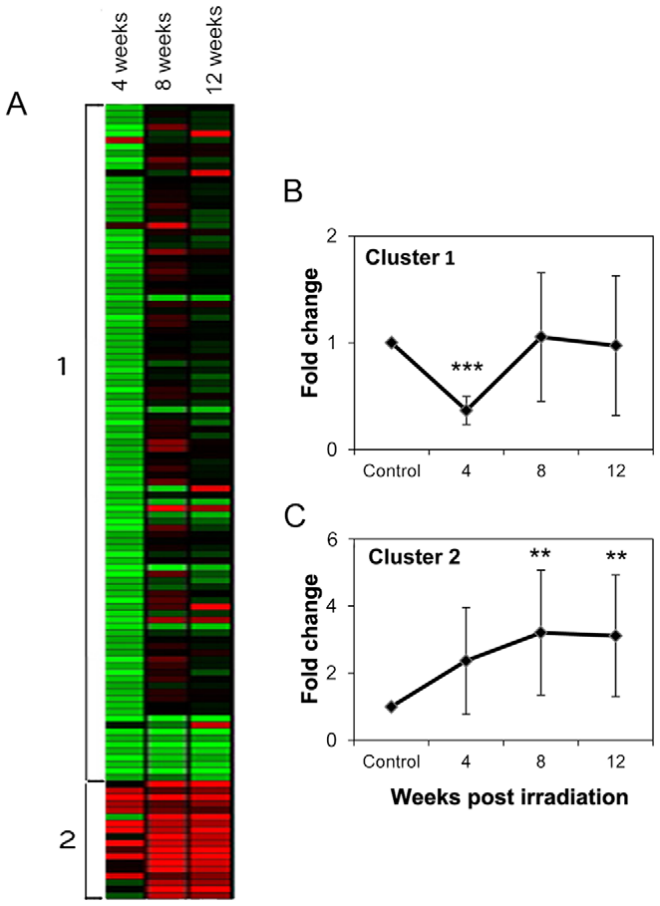
Transcriptomic expression patterns following 15-Gy irradiation. A. Expression profiles of differentially expressed genes (n = 3) 4, 8 and 12 weeks post-irradiation depicted by Heatmap and Expender analysis and grouped into two clusters based on temporal expression profiles: genes with up-regulated expression are shown in red, and those with down-regulated expression are shown in green. B. 81 genes were down-regulated at least twofold 4 weeks post-irradiation, and recovered to around control levels 8 and 12 weeks post-irradiation. *** *P*<0.0001 for 4-week values vs. control, 8- and 12-week post-irradiation values. C. 14 genes were twofold up-regulated 4 weeks post-irradiation, and further increased threefold 8 and 12 weeks post-irradiation. **P<0.001 for control vs. all 8- and 12-week post-irradiation values.

**Figure 3:**
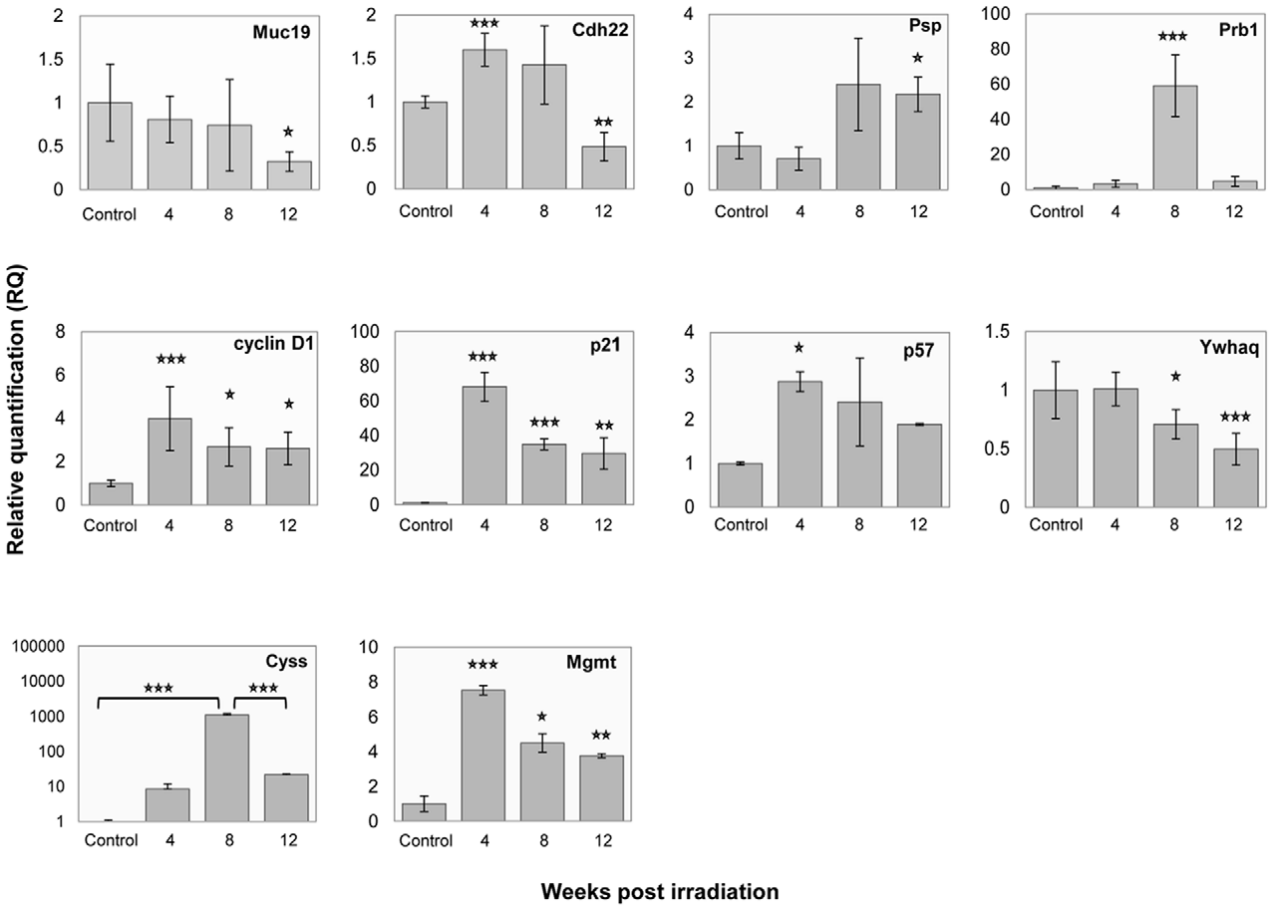
Fold change in gene expression following irradiation. Relative quantification (RQ) values of Muc19, Cdh22, Psp, Prb1, CysS, Mgmt, cyclin d1, p21, p57 and Ywhaq gene expression during the 12 weeks post-irradiation. Validation of microarray values for salivary gland functional gene expression following irradiation by TaqMan real-time PCR. The data are expressed as means of n = 3. *P<0.05, **P<0.005, ***P<0.001 compared to control levels, unless otherwise stated.

Following IR, two major expression patterns were identified among the differentially expressed genes (Fig. 2A). The first included 81 genes that were down-regulated at least twofold 4 weeks post-IR, and recovered to near control levels 8 and 12 weeks post-IR (Fig. 2B). The second pattern included 14 genes which were up-regulated following IR and then continued to be over-expressed during the 12-week period following IR (Fig. 2C). Within the first pattern, down-regulated (compared to controls) SG-expressed genes included those encoding mucin 19 (Muc19), cadherin 22 (Cdh22), parotid secretory protein (Psp) and proline-rich protein of BstNI subfamily 1 (Prb1), as validated by Taqman real-time PCR (Fig. 3). Muc19 and Cdh22 genes showed ca. twofold down-regulation 12 weeks post-IR. Psp gene showed the same level of down-regulation at week 4 post-IR, and was then up-regulated ca. twofold (compared to control levels) at 8 and 12 weeks post-IR (Fig. 3). Contrary to the microarray results, Prb1 gene was found by real-time PCR to be up-regulated following IR and therefore actually belonged to the second pattern.

The second expression pattern demonstrated an at least twofold up-regulation in gene expression following IR (Fig. 2C), and included the remaining 14 genes: cell-cycle regulating genes, and those encoding Mgmt (O-6-methylguanine-DNA methyltransferase), CysS and α-amylase. The latter two genes encode major salivary secretory proteins and were up-regulated from week 4 post-IR. The CysS gene was up-regulated following IR to a maximum 1108-fold increase at 8 weeks post-IR and subsided to a 21-fold higher level than controls at 12 weeks post-IR (Fig. 3). Among the cell-cycle pathway genes, four were found to be affected by IR during the 12-week timeframe: cyclin D1 gene, *p21* (encoding cyclin-dependent kinase [CDK] inhibitor 1A), *p57* (encoding CDK inhibitor 1C) and *Ywhaq* (encoding tyrosine 3-monooxygenase/tryptophan 5-monooxygenase activation protein, theta polypeptide). Since changes in these genes are associated with cell-cycle arrest, their expressions were validated by real-time PCR. As shown in Fig. 3, cyclin D1 gene was up-regulated fourfold 4 weeks post-IR and remained ∼2.5-fold up-regulated 8 and 12 weeks post-IR. *p21* was up-regulated 68-fold at 4 weeks post-IR and remained high at 8 weeks (35-fold) and 12 weeks (30-fold) post-IR. *p57* was up-regulated 2.8- to 1.9-fold following IR. *Ywhaq*, encoding a 14-3-3 family member, was only down-regulated twofold at 12 weeks post-IR.

Interestingly, the large decrease in the transcription of salivary proteins in the first pattern and the increased expression of cell-cycle-regulating genes in the second pattern were accompanied by increased expression of the gene encoding Mgmt, a DNA repair enzyme, following IR (see below in pattern 2). This gene was analyzed by real-time PCR and exhibited a 7.5-fold increase at 4 weeks, a decrease to 4.5-fold at 8 weeks post-IR, and then remained at a 3.7-fold higher level than controls at 12 weeks post-IR (Fig. 3).

The genes encoding CysS and Psp showed the same expression pattern as in the proteomic analysis, i.e. CysS transcription was found to be up-regulated in the mRNA analysis by Taqman (Fig. 3) and in spots 14 and 15 by proteomic analysis. Psp transcription was slightly down-regulated in the mRNA analysis by Taqman (Fig. 3) 4 weeks post-IR, and in the proteomic analysis it was found in several spots: 1, 3, 5, 6 and 8; although the behavior differed slightly for each spot, the prominent trend was down-regulation (Table S1). In the mRNA analysis, expression level was up-regulated at 8 and 12 weeks, but in the proteomic analysis, the damage continued and no return to normal levels was observed.

**Figure 4:**
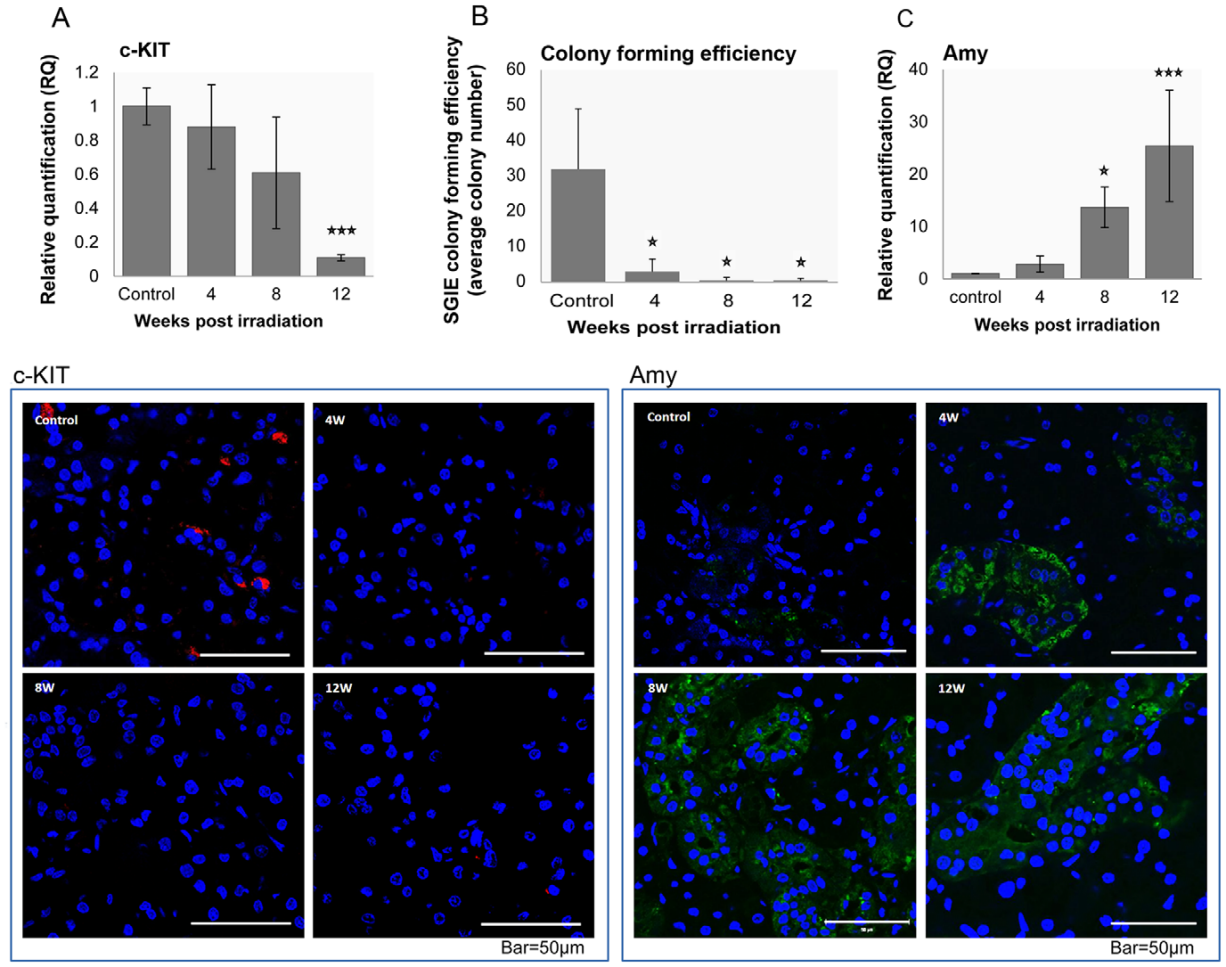
Gene expression, SGIE colony forming efficiency and immunofluorescence staining during 12 weeks post-irradiation. A. In irradiated glands, c-KIT expression decreases progressively (4W, 8W, 12W) compared to control glands. Immunofluorescence staining shows a similar pattern (bar = 50 µm). B. SGIE colony forming efficiency in control and 4, 8 and 12-week post-IR. The number of rats used in each experiment is indicated inside the bars. *Significantly different at P<0.02. C. α-amylase gene expression is increased (4W, 8W, 12W) compared to control glands. Immunofluorescence staining shows a similar pattern (bar = 50 µm). Data are expressed as means of n = 3. *P<0.05, ***P<0.001.

We also measured the effect of IR on the c-KIT salivary cell proliferating fraction by real-time PCR (Fig. 4A). c-KIT expression showed a 10-fold reduction 12 weeks post-IR. We validated these results using confocal microscopy, observing the same decrease in expression pattern (Fig. 4A). To further validate cell proliferation/cell arrest data in another independent approach we counted the number of SGIE cell colonies obtained from the submandibular salivary glands before and 4, 8 and 12 weeks after irradiation (Fig. 4B). The average number of grown colonies per animal without irradiation was 32±17, while 4 weeks after head and neck irradiation, SGIE colony growth was 10 times lower, and about 75 lower (compared to control) 8 and 12 weeks post irradiation (0.5±0.5).

As normally, α-amylase is not produced by the SSG [6], we validated these results by Taqman real-time PCR. We found a gradual time-dependent increase in α-amylase transcription from control levels in non-irradiated animals to 13-fold and 25-fold at 8 and 12 weeks post-IR, respectively (Fig. 4C). In contrast, α-amylase protein expression exhibited up-regulation following IR in protein spots 15 and 18 (Table S1), in agreement with the mRNA analysis (Fig. 4C), but in the other α-amylase protein spots it was down-regulated (spots 1, 11). We further validated these results using confocal microscopy which showed the same increase in expression pattern (Fig. 4C).

## Discussion

IR therapy for head and neck cancer patients results in irreversible damage to the salivary parenchymal tissue. In the last decade, attempts have been made to decrease IR damage to the SG using a variety of approaches that can be grouped into three categories: (i) physical approaches such as intensity-modulated radiation therapy and 3-D radiotherapy [25]; (ii) pharmacological approaches such as exploring the usefulness of sialogogues or radical scavengers to protect glands from IR damage; (iii) biological approaches such as gene transfer [26], and tissue- and cell-regeneration methodologies [4], [15], [27], [28]. However to date, the pathogenesis underlying the irreversible damage has not been adequately explored and many questions remain to be addressed [2]. The aim of this study was to analyze proteomic and transcriptomic changes in saliva following IR of SSG in a rodent model. We hypothesized that understanding the global mechanistic changes that occur following IR will eventually allow us to promote biological treatment modalities for these patients. The IR therapy used on human patients is a fractionated dose [29]. However, in experimental animal models, it has been shown that a single high dose or its equivalent dose as fractionated IR lead to essentially the same end result, i.e. reduced saliva secretion [6], [7]. We therefore used a single dose in our experiments. In an initial confirmation of previous experiments, we found that a single 15-Gy dose of IR to the head and neck in a rat model reduces saliva output to 50% of normal [12], [30] as well as inducing minimal salivary tissue damage with no signs of fibrosis [2], [11], [12].

To better understand the mechanisms underlying salivary gland IR damage, we performed both a proteomic analysis of the saliva and gene array of the SSG tissue before and after IR.

As IR affects protein production, we examined its global changing effect on the saliva proteome by comparing it to non-irradiated animals and to previous reported proteomic maps [31], [32], [33], [34]. Proteomic analysis of oral fluid (whole saliva) protein composition represents the summed effect of the IR treatment on all glands, not only on the SSGs. It reflects at least two different consequences of the IR: (i) reduced salivary-originated proteins and (ii) increased serum-derived proteins. The reduction in salivary proteins in the oral fluids occurred in two steps and became larger with time. Four weeks after IR, expression of most proteins decreased, followed by partial recovery at week 8 and a further decrease at week 12 post-IR. Comparison of these results to the gene-array assay showed partial similarity at the 4-week time point (pattern 1, Fig. 2B); however at the later time points, while gene expression recovered, protein levels remained low. This decrease in protein synthesis might be explained by the transcriptomic data showing damage to protein synthesis, processing or trafficking pathways (pattern 1). The increase in serum proteins, mainly albumin, in the saliva indicates damage to the tight junctions in the salivary cell membrane causing loss of barrier function between serum and secretory ductal glandular compartment components. Tight junctions are also needed for directional fluid movement from the acini to the ducts and therefore, their damage may contribute to a reduction in the efficiency of fluid secretion by the glands. These findings are also supported by our real-time PCR results showing down-regulation of cell-adhesion molecules such as Cdh22, and by our microarray results showing down-regulation of genes encoding epithelial cell-adhesion molecule (Epcam) and gap junction beta 2 (Gjb2).

Gene-array analyses showed that following IR, the expression of 95 genes was modified following two general patterns. The most prominent pattern, shown by 81 of the genes, was a damage-like response. The genes exhibiting this pattern were grouped into categories associated mainly with protein synthesis and secretion (endoplasmic reticulum, protein transport, golgi apparatus, protein folding and secretory granule), indicating a reduction in the cell's ability to produce and secrete protein products not only as a direct result of possibly lower transcription levels but also as a result of damage to protein synthesis, processing or trafficking pathways. To gain a better understanding of IR damage to SG functionality, results from several of the SG-derived genes (encoding Muc19, Cdh22, Psp, α-amylase and CysS), a DNA repair gene (encoding Mgmt) and proliferating genes (encoding c-KIT) were validated using Taqman real-time PCR (Fig. 3).

The main functions of mucus include lubrication and protection of epithelia from environmental insults. The gel-like properties of mucus are generally attributed to the physical properties and structural features of the gel-forming mucins, such as Muc19 [35]. Muc19 was down-regulated twofold 12 weeks post-IR, which might cause the saliva to lose its lubricating and protective properties, and contribute to the xerostomia [3] seen in irradiated patients. Cdh22 belongs to the cadherins, a superfamily of transmembrane glycoproteins which typically mediate calcium-dependent homophilic intercellular adhesion [36]. Cdh22 was found to be downregulated twofold 12 weeks post-IR, indicating damage in signaling and adhesion. Psp, a proacinar cell marker, is associated with rat SSG regeneration [37]: in the adult regenerating gland, mRNA levels of Psp have been found to be up-regulated compared to ligated glands [37]. The increased expression of Psp 12 weeks post-IR suggests that the salivary glands are attempting to regenerate (see also our discussion on cell-cycle genes below).

Saliva contains a prominent family of proline-rich proteins (PRPs) which includes acidic, basic and glycosylated PRPs [38]. Interestingly, ∼60-fold up-regulation of Prb1 was observed following IR (Fig. 3). The salivary PRPs have several important activities: they bind hydroxyapatite and calcium, mediate adherence of microorganisms, and modify the lubricative properties of saliva. The Prb1 gene encodes a precursor of basic proline-rich salivary proteins and is synthesized mainly in the acinar cells [39]. CysS belongs to the cystatins, natural inhibitors of cysteine proteinases through their tight binding to the proteinases' active part [40]. CysS is expressed in the acinar cells of the SG [40] and has been reported to play a role in the response to injury [40]. The up-regulation in CysS mRNA levels following IR suggests an attempt to deal with the damage caused to the salivary glands. The results for submandibular expression of α-amylase, i.e. induced expression of its gene and protein, was not supported by the 2-DE analysis of whole saliva. This difference could be either genuine i.e., the net effect on the saliva secreted by all glands is a reduction in α-amylase protein, or it could be of technical origin. In a typical 2-DE gel, about 140 α-amylase protein spots are identified [41], but only a few of them were taken for analysis in this proteomic study, preventing an accurate quantitative interpretation. A further quantitative proteomic approach, such as dimethylation based quantitative activity assay, is suggested to clarify this point.

Multiple pathways are involved in the maintenance of genetic integrity after exposure to ionizing radiation, most of which are related to the cell cycle [42]. Cells commonly respond to DNA-damaging agents by activating cell-cycle checkpoints. These checkpoints provide controlled temporary arrest at a specific stage of the cell cycle to allow the cell to correct possible defects [43]. Interestingly, four cell-cycle regulatory genes: cyclin D1 gene, *p21*, *p57* and *Ywhaq*, were affected by IR. The first three genes shared the same pattern of expression following IR: their major differential up-regulation occurred 4 weeks post-IR, and it was slightly reduced toward 12 weeks post-IR (second pattern). *Ywhaq* was only down-regulated at 12 weeks post-IR.

Damage to the cells' proliferation ability might also be a result of direct DNA damage, as seen by the increased transcription of Mgmt (second pattern) following IR. Mgmt is a suicide repair enzyme that removes a methyl group from the O^6^ position in guanine, thus inactivating itself while repairing the DNA [44]. Thus, Mgmt plays an important role in maintaining genomic integrity when DNA damage occurs. The effect of IR on cell-regulation genes as well as on Mgmt and SG-function genes supports the hypothesis that IR results in signaling damage combined with DNA damage [2], [12], [13].

Because of the complex cell processes determining cell proliferation and cell-cycle arrest, we also examined the outcome of this balance by monitoring in-vivo c-KIT transcription, c-KIT tissue expression and number of grown salivary cell colonies following IR.

c-KIT-expressing cells have been suggested to belong to the SG cell-dividing fraction [15] as well as SGIE cells which were reported to be able to grow in vitro for 20 passages [45]. Indeed, IR reduced c-KIT expression as well as number of SGIE colonies, 10 and 75 fold at 12 weeks post-IR respectively (Fig.4A, 4B) exemplifying cell arrest transcriptomic data. Collectively, our data on the expression patterns of cell-cycle genes, c-KIT and colony forming efficiency suggest that a cell-cycle arrest response occurs mainly at 4 to 8 weeks post-IR.

In summary, this study emphasizes the complexity manifested by the multifactorial molecular mechanisms involved in SG damage post IR. It demonstrates that IR damages the cells' abilities to produce and secrete fluids and proteins, and maintain the physiological barrier between serum and saliva. As shown herein, the mechanisms governing this damage involve the cells' protein synthesis and secretion machineries. This damage is irreversible because tissue-regeneration ability is also damaged. Cells are damaged by the IR and undergo cell-cycle arrest as seen by the up-regulation of CDK inhibitors and down-regulation of *Ywhaq*. The data presented in this study can provide treatment targets for cell- and gene-therapy approaches, as well as a baseline to follow up on treatment success by comparing cellular, transcriptomic and proteomic characteristics before and after treatment. As rodents salivary glands respond to irradiation in a different way than humans [2], further human studies should be undertaken to support these findings and currently we analyze the protein profile changes in oral fluids of ^131^I treated thyroid cancer patients, irradiation treated head and neck cancer patients and normal healthy subjects.

## Supporting Information

Table S1 **MS identification of selected spots.**
(DOC)

Table S2 **Differentially expressed genes 4, 8 and 12 weeks post-irradiation.**
(DOC)
