# Supplementary material for: Effect of Irradiation on Cell Transcriptome and Proteome of Rat Submandibular Salivary Glands
Source: PLoS One. 2012 Jul 6;7(7):e40636. doi: 10.1371/journal.pone.0040636 (PMC3391292; doi:10.1371/journal.pone.0040636)
Supplement: Table S1 — MS identification of selected spots. (DOC) [file pone.0040636.s001.doc]

Table S1 - MS identification of selected spots

| **spot** | **protein identification** | **accesion no.** | **theor. MW** | **theor. pI** | **matched peptides** | **Sequence coverage (%)** |
| --- | --- | --- | --- | --- | --- | --- |
| 1 | Alpha-amylase 1 precursor | Q5I0L0 | 57501 | 5.97 | 4 | 8.40 |
|  | Parotid secretory protein precursor | Q63471 | 24514 | 4.68 | 3 | 17.00 |
| 2 | Cysteine-rich secretory protein 1 precursor | P12020 | 27829 | 4.87 | 5 | 16.26 |
| 3 | Von Ebner gland protein 1 precursor | P20289 | 19713 | 6.09 | 10 | 38.42 |
|  | Cysteine-rich secretory protein 1 precursor | P12020 | 27829 | 4.87 | 9 | 27.24 |
|  | Ig kappa chain C region, A allele | P01836 | 11725 | 4.99 | 6 | 50.94 |
|  | Ig lambda-2 chain C region | P20767 | 11311 | 5.76 | 5 | 64.42 |
|  | Adenosine deaminase | Q920P6 | 39874 | 5.32 | 5 | 11.08 |
|  | Von Ebner gland protein 2 precursor | P41244 | 19687 | 6.09 | 4 | 27.12 |
|  | Parotid secretory protein precursor | Q63471 | 24514 | 4.68 | 4 | 18.72 |
| 4 | Parotid secretory protein precursor | Q63471 | 24514 | 4.68 | 6 | 20.90 |
|  | Odorant-binding protein precursor | P08937 | 19687 | 5.32 | 3 | 23.30 |
| 5 | Parotid secretory protein precursor | Q63471 | 24514 | 4.68 | 8 | 28.51 |
|  | Cysteine-rich secretory protein 1 precursor | P12020 | 27829 | 4.87 | 8 | 24.39 |
|  | Keratin, type I KA16 | Q6IFU9 | 50777 | 5.05 | 8 | 22.62 |
|  | Vimentin | P48616 | 53695 | 5.06 | 6 | 13.09 |
|  | Von Ebner gland protein 1 precursor | P20289 | 19713 | 6.09 | 5 | 25.42 |
|  | Prolactin-inducible protein homolog precursor | O70417 | 16428 | 4.58 | 5 | 17.12 |
| 6 | Odorant-binding protein precursor | P08937 | 19687 | 5.32 | 8 | 48.84 |
|  | Cysteine-rich secretory protein 1 precursor | P12020 | 27829 | 4.87 | 8 | 25.61 |
|  | Von Ebner gland protein 1 precursor | P20289 | 19713 | 6.09 | 7 | 45.20 |
|  | Parotid secretory protein precursor | Q63471 | 24514 | 4.68 | 7 | 26.38 |
|  | Prolactin-inducible protein homolog precursor | O70417 | 16428 | 4.58 | 6 | 17.12 |
|  | Von Ebner gland protein 2 precursor | P41244 | 19687 | 6.09 | 5 | 27.12 |
| 7 | Odorant-binding protein precursor | P08937 | 19687 | 5.32 | 14 | 49.70 |
|  | Prolactin-inducible protein homolog precursor | O70417 | 16428 | 4.58 | 11 | 52.90 |
|  | Von Ebner gland protein 1 precursor | P20289 | 19713 | 6.09 | 7 | 22.00 |
|  | Apolipoprotein E precursor | P02650 | 35731 | 5.23 | 6 | 17.50 |
| 8 | Cysteine-rich secretory protein 1 precursor | P12020 | 27829 | 4.87 | 11 | 32.93 |
|  | Parotid secretory protein precursor | Q63471 | 24514 | 4.68 | 9 | 28.51 |
|  | Apolipoprotein A-IV precursor | P02651 | 44429 | 5.12 | 8 | 19.69 |
|  | Prolactin-inducible protein homolog precursor | O70417 | 16428 | 4.58 | 6 | 17.12 |
|  | Odorant-binding protein precursor | P08937 | 19687 | 5.32 | 5 | 27.91 |
|  | Von Ebner gland protein 1 precursor | P20289 | 19713 | 6.09 | 5 | 25.42 |
|  | Deoxyribonuclease-1 precursor | P21704 | 32044 | 5.06 | 5 | 20.07 |
|  | Apolipoprotein A-I precursor | P04639 | 30043 | 5.52 | 5 | 18.15 |
| 9 | Serotransferrin precursor | P12346 | 76346 | 7.14 | 31 | 29.66 |
| 10 | Serum albumin precursor | P02770 | 68686 | 6.09 | 9 | 15.30 |
|  | Fetuin-B precursor | Q9QX79 | 41506 | 6.71 | 5 | 10.80 |
| 11 | Vitamin D-binding protein precursor | P04276 | 53509 | 5.65 | 14 | 20.80 |
|  | Alpha-amylase 1 precursor | Q5I0L0 | 57501 | 5.97 | 14 | 15.26 |
|  | Long palate, lung and nasal epithelium carcinoma-associated protein 1 precursor | A0JPN3 | 52219 | 5.80 | 13 | 20.46 |
|  | Cysteine-rich secretory protein 1 precursor | P12020 | 27829 | 4.87 | 12 | 34.15 |
|  | Alpha-amylase 1 precursor | Q5I0L0 | 57501 | 5.97 | 11 | 14.48 |
|  | Angiotensinogen precursor | P01015 | 51949 | 5.37 | 9 | 18.45 |
|  | Fetuin-B precursor | Q9QX79 | 41506 | 6.71 | 9 | 18.25 |
|  | T-kininogen 1 precursor | P01048 | 47745 | 6.08 | 8 | 16.74 |
| 12 | Serine protease inhibitor A3N precursor | P09006 | 46622 | 5.32 | 20 | 37.08 |
|  | Alpha-amylase 1 precursor | Q5I0L0 | 57501 | 5.97 | 19 | 17.03 |
|  | Serine protease inhibitor A3K precursor | P05545 | 46532 | 5.31 | 18 | 37.02 |
|  | Alpha-1-antiproteinase precursor | P17475 | 46107 | 5.70 | 16 | 33.82 |
|  | Serine protease inhibitor A3M precursor | Q63556 | 46066 | 5.65 | 15 | 29.61 |
|  | Serum albumin precursor | P02770 | 68686 | 6.09 | 15 | 27.80 |
|  | Serine protease inhibitor A3L precursor | P05544 | 46248 | 5.48 | 14 | 28.81 |
|  | Alpha-1-inhibitor 3 precursor | P14046 | 163669 | 5.70 | 13 | 9.00 |
|  | Long palate, lung and nasal epithelium carcinoma-associated protein 1 precursor | A0JPN3 | 52219 | 5.80 | 11 | 18.78 |
|  | Vitamin D-binding protein precursor | P04276 | 53509 | 5.65 | 11 | 18.28 |
|  | Protein disulfide-isomerase precursor | P04785 | 56951 | 4.82 | 10 | 18.04 |
|  | Serotransferrin precursor | P12346 | 76346 | 7.14 | 10 | 15.62 |
|  | Cysteine-rich secretory protein 1 precursor | P12020 | 27829 | 4.87 | 8 | 30.49 |
| 13 | Long palate, lung and nasal epithelium carcinoma-associated protein 1 precursor | A0JPN3 | 52219 | 5.80 | 12 | 20.25 |
|  | Serum albumin precursor | P02770 | 68686 | 6.09 | 11 | 18.09 |
|  | Alpha-amylase 1 precursor | Q5I0L0 | 57501 | 5.97 | 11 | 15.66 |
|  | Fetuin-B precursor | Q9QX79 | 41506 | 6.71 | 9 | 10.18 |
| 14 | Serum albumin precursor | P02770 | 68686 | 6.09 | 35 | 41.28 |
|  | Cystatin-S precursor | P19313 | 15939 | 4.76 | 4 | 27.66 |
| 15 | Cystatin-S precursor | P19313 | 15939 | 4.76 | 9 | 39.00 |
|  | Apolipoprotein E precursor | P02650 | 35753 | 5.23 | 8 | 14.80 |
|  | Cystatin-A | P01039 | 11556 | 5.42 | 6 | 43.70 |
|  | Haptoglobin precursor | P06866 | 38539 | 6.10 | 5 | 16.70 |
|  | Prolactin-inducible protein homolog precursor | O70417 | 16428 | 4.58 | 4 | 24.00 |
|  | Parotid secretory protein precursor | Q63471 | 24514 | 4.68 | 4 | 23.40 |
| 16 | Serum albumin precursor | P02770 | 68686 | 6.09 | 24 | 34.70 |
|  | Complement C3 precursor | P01026 | 186342 | 6.12 | 12 | 8.24 |
|  | Complement C4 precursor | P08649 | 192042 | 6.99 | 12 | 6.74 |
|  | Serotransferrin precursor | P12346 | 76346 | 7.14 | 11 | 16.19 |
|  | Hemopexin precursor | P20059 | 51351 | 7.58 | 9 | 17.61 |
|  | Vomeromodulin | Q63751 | 10884 | 8.19 | 8 | 68.37 |
| 17 | Serum albumin precursor | P02770 | 68686 | 6.09 | 28 | 34.70 |
|  | Serotransferrin precursor | P12346 | 76346 | 7.14 | 13 | 14.76 |
|  | Complement C3 precursor | P01026 | 186342 | 6.12 | 13 | 6.92 |
|  | Vomeromodulin | Q63751 | 10884 | 8.19 | 10 | 71.43 |
|  | T-kininogen 1 precursor | P01048 | 47745 | 6.08 | 9 | 17.44 |
|  | Hemopexin precursor | Q91X72 | 51351 | 7.58 | 8 | 17.61 |
|  | Complement C4 precursor | P08649 | 192042 | 6.99 | 7 | 3.91 |
| 18 | Alpha-amylase 1 precursor | Q5I0L0 | 57501 | 5.97 | 24 | 26.22 |
|  | Serotransferrin precursor | P12346 | 76346 | 7.14 | 16 | 18.34 |
|  | Serum albumin precursor | P02770 | 68686 | 6.09 | 13 | 23.36 |
|  | Long palate, lung and nasal epithelium carcinoma-associated protein 1 precursor | A0JPN3 | 52219 | 5.80 | 9 | 15.40 |
|  | Ig gamma-2A chain C region | P20760 | 35163 | 7.72 | 8 | 22.67 |
| 19 | Serum albumin precursor | P02770 | 68686 | 6.09 | 9 | 14.10 |
|  | Alpha-1-inhibitor 3 precursor | P14046 | 163669 | 5.70 | 7 | 3.90 |
| 20 | Cysteine-rich secretory protein 1 precursor | P12020 | 27829 | 4.87 | 19 | 34.55 |
|  | Deoxyribonuclease-1 precursor | P21704 | 32044 | 5.06 | 11 | 41.20 |
|  | Apolipoprotein E precursor | P02650 | 35753 | 5.23 | 9 | 22.19 |
|  | Parotid secretory protein precursor | Q63471 | 24514 | 4.68 | 4 | 20.00 |
| 21 | Polymeric immunoglobulin receptor precursor | P15083 | 84745 | 5.07 | 26 | 23.10 |
|  | Serotransferrin precursor | P12346 | 76346 | 7.14 | 24 | 22.80 |
|  | Afamin precursor | P36953 | 69290 | 5.87 | 16 | 23.40 |
|  | Alpha-1B-glycoprotein precursor | Q9EPH1 | 56443 | 6.89 | 16 | 22.40 |
|  | Serum albumin precursor | P02770 | 68686 | 6.09 | 15 | 21.20 |
|  | Alpha-1-inhibitor 3 precursor | P14046 | 163669 | 5.70 | 15 | 10.40 |
|  | Alpha-1-macroglobulin precursor | Q63041 | 167019 | 6.46 | 15 | 10.00 |
|  | Cysteine-rich secretory protein 1 precursor | P12020 | 27829 | 4.87 | 11 | 29.70 |
|  | Plasma protease C1 inhibitor precursor | Q6P734 | 55576 | 5.53 | 11 | 20.60 |
|  | Complement C3 precursor | P01026 | 186342 | 6.12 | 10 | 6.60 |
| 22 | Alpha-1-macroglobulin precursor | Q63041 | 167019 | 6.46 | 28 | 11.67 |
|  | Alpha-1-inhibitor 3 precursor | P14046 | 163669 | 5.70 | 21 | 15.64 |
|  | Serum albumin precursor | P02770 | 68686 | 6.09 | 20 | 28.13 |
|  | Complement C3 precursor | P01026 | 186342 | 6.12 | 20 | 15.27 |
|  | T-kininogen 1 precursor | P01048 | 47745 | 6.08 | 15 | 28.14 |
|  | Complement component C9 precursor | Q62930 | 62241 | 5.51 | 15 | 24.01 |
|  | Vomeromodulin | Q63751 | 10884 | 8.19 | 13 | 71.43 |
|  | Cysteine-rich secretory protein 1 precursor | P12020 | 27829 | 4.87 | 13 | 30.89 |
| 23 | Corticosteroid-binding globulin precursor | P31211 | 44643 | 4.80 | 11 | 13.38 |
|  | Serum albumin precursor | P02770 | 68686 | 6.09 | 10 | 18.42 |
|  | Fetuin-B precursor | Q9QX79 | 41506 | 6.71 | 8 | 18.25 |
|  | Serotransferrin precursor | P12346 | 76346 | 7.14 | 8 | 11.75 |
|  | Alpha-1-antiproteinase precursor | P17475 | 46107 | 5.70 | 7 | 14.11 |
|  | Cysteine-rich secretory protein 1 precursor | P12020 | 27829 | 4.87 | 6 | 21.54 |
|  | Angiotensinogen precursor | P01015 | 51949 | 5.37 | 6 | 15.30 |
| 24 | Complement C3 precursor | P01026 | 186342 | 6.12 | 16 | 10.70 |
|  | Cysteine-rich secretory protein 1 precursor | P12020 | 27829 | 4.87 | 14 | 32.93 |
|  | Fibrinogen beta chain precursor | P14480 | 54201 | 7.89 | 13 | 18.16 |
|  | Serum albumin precursor | P02770 | 68686 | 6.09 | 12 | 20.89 |
|  | Alpha-1-macroglobulin precursor | Q63041 | 167019 | 6.46 | 12 | 9.00 |
|  | Polymeric immunoglobulin receptor precursor | P15083 | 84745 | 5.07 | 11 | 21.59 |
|  | Transcobalamin-2 precursor | Q9R0D6 | 47390 | 7.63 | 10 | 25.76 |
|  | Deoxyribonuclease-1 precursor | P21704 | 32044 | 5.06 | 9 | 30.28 |
|  | Alpha-1-inhibitor 3 precursor | P14046 | 163669 | 5.70 | 9 | 7.18 |
|  | Apolipoprotein A-IV precursor | P02651 | 44429 | 5.12 | 8 | 25.06 |
|  | Adenosine deaminase | Q920P6 | 39874 | 5.32 | 8 | 25.00 |
|  | Serine protease inhibitor A3N precursor | P09006 | 46622 | 5.32 | 8 | 22.49 |
|  | Prolactin-inducible protein homolog precursor | O70417 | 16428 | 4.58 | 7 | 30.14 |
| 25 | Serum albumin precursor | P02770 | 68686 | 6.09 | 15 | 22.20 |
|  | Alpha-1-macroglobulin precursor | Q63041 | 167019 | 6.46 | 12 | 7.20 |
|  | Fibrinogen beta chain precursor | P14480 | 54201 | 7.89 | 7 | 14.41 |
|  | Proline-rich proteoglycan 2 precursor | P10165 | 30008 | 5.94 | 6 | 54.24 |
|  | Cysteine-rich secretory protein 1 precurso | P12020 | 27829 | 4.87 | 6 | 21.54 |
|  | Apolipoprotein A-IV precursor | P02651 | 44429 | 5.12 | 6 | 17.90 |
|  | Complement C3 precursor | P01026 | 186342 | 6.12 | 5 | 3.55 |
| 26 | Serum albumin precursor | P02770 | 68686 | 6.09 | 9 | 16.28 |
|  | Fetuin-B precursor | Q9QX79 | 41506 | 6.71 | 7 | 15.34 |
|  | Cysteine-rich secretory protein 1 precursor | P12020 | 27829 | 4.87 | 6 | 25.61 |
|  | Serotransferrin precursor | P12346 | 76346 | 7.14 | 6 | 10.03 |
|  | Alpha-1-antiproteinase precursor | P17475 | 46107 | 5.70 | 5 | 12.90 |
|  | Corticosteroid-binding globulin precursor | P31211 | 44643 | 4.80 | 5 | 9.85 |
