# Supplementary material for: Effect of Irradiation on Cell Transcriptome and Proteome of Rat Submandibular Salivary Glands
Source: PLoS One. 2012 Jul 6;7(7):e40636. doi: 10.1371/journal.pone.0040636 (PMC3391292; doi:10.1371/journal.pone.0040636)
Supplement: Table S2 — Differentially expressed genes 4, 8 and 12 weeks post-irradiation. (DOC) [file pone.0040636.s002.doc]

Table S2.

Differentially expressed genes before, 4, 8 and 12 weeks post irradiation

| **Gene symbol** | **Accession number** | **Description and function** | **Gene symbol** | **Accession number** | **Description and function** |
| --- | --- | --- | --- | --- | --- |
| Acsl1 | NM_012820 | cyl-CoA synthetase long-chain family member 1 | Mgmt | NM_012861 | O-6-methylguanine-DNA methyltransferase |
| Agr2 | NM_001106725 | anterior gradient homolog 2 (Xenopus laevis) | Muc19 | XM_235593 | mucin 19 |
| Alg5 | NM_001025407 | asparagine-linked glycosylation 5, dolichyl-phosphate beta-glucosyltransferase homolog | Ndfip2 | NM_001108390 | Nedd4 family interacting protein 2 |
| Amy1a | NM_001010970 | amylase, alpha 1A (salivary) | Ndp | NM_001108814 | Norrie disease (pseudoglioma) (human) |
| Aph1b | XM_217185 | anterior pharynx defective 1 homolog B (C. elegans) | Ndufs1 | NM_001005550 | NADH dehydrogenase (ubiquinone) Fe-S protein 1 |
| Arf4 | NM_024151 | ADP-ribosylation factor 4 | Ntf4 | NM_013184 | neurotrophin 4 |
| Arl1 | NM_022385 | ADP-ribosylation factor-like 1 | Odc1 | NM_012615 | ornithine decarboxylase 1 |
| Arntl | NM_024362 | aryl hydrocarbon receptor nuclear translocator-like | Pdia6 | NM_001004442 | protein disulfide isomerase associated 6 |
| Atp5a1 | NM_023093 | ATP synthase, H+ transporting, mitochondrial F1 complex, alpha subunit 1, cardiac muscle | Pf4 | NM_001007729 | platelet factor 4 |
| Atp6ap2 | XM_217592 | ATPase, H+ transporting, lysosomal accessory protein 2 | Pgam1 | NM_053290 | phosphoglycerate mutase 1 (brain) |
| Auh | NM_001108407 | AU RNA binding protein/enoyl-coenzyme A hydratase | Pgcp | NM_031640 | plasma glutamate carboxypeptidase |
| Bcas1 | NM_145670 | breast carcinoma amplified sequence 1 | Pon2 | NM_001013082 | paraoxonase 2 |
| Bhlhe41 | NM_133303 | basic helix-loop-helix family, member e41 | Ppapdc1b | NM_001109411 | phosphatidic acid phosphatase type 2 domain containing 1B |
| Bpifb2 | NM_001106531 | BPI fold containing family B, member 2 | Prb1 | NM_172065 | proline-rich protein BstNI subfamily 1 |
| Ccnd1 | NM_171992 | cyclin D1 | Psmd14 | XM_001059730 | proteasome (prosome, macropain) 26S subunit, non-ATPase, 14 |
| Cd164 | NM_031812 | CD164 antigen | Psp | NM_052808 | parotid secretory protein |
| Cdh22 | NM_019161 | cadherin 22 | Rab1 | NM_031090 | RAB1, member RAS oncogene family |
| Cdkn1a | NM_080782 | cyclin-dependent kinase inhibitor 1A (p21) | Rap1a | NM_001005765 | RAP1A, member of RAS oncogene family |
| Cdkn1c | NM_001033757 | cyclin-dependent kinase inhibitor 1C (p57) | Rasl12 | NM_001108162 | RAS-like, family 12 |
| Cept1 | NM_001007699 | choline/ethanolamine phosphotransferase 1 | Rbbp7 | NM_031816 | retinoblastoma binding protein 7 |
| Chia | NM_207586 | chitinase, acidic | RGD1559532 | NM_001024983 | salivary proline-rich protein |
| Chpt1 | NM_001007750 | choline phosphotransferase 1 | RGD1560483 | XM_231013 | similar to Cystatin S precursor (LM protein) |
| Cmah | NM_001024273 | cytidine monophosphate-N-acetylneuraminic acid hydroxylase (CMP-N-acetylneuraminate monooxygenase) pseudogene | RGD1565709 | XM_342764 | similar to ovostatin-2 |
| Cpd | NM_012836 | carboxypeptidase D | Sar1b | NM_001009622 | GTP-binding protein SAR1b |
| Cthrc1 | NM_172333 | collagen triple helix repeat containing 1 | Scgb3a1 | NM_001013180 | secretoglobin, family 3A, member 1 |
| Cyss | NM_198685 | cystatin S | Sec23b | NM_001108593 | Sec23 homolog B (S. cerevisiae) |
| Dbp | NM_012543 | D site of albumin promoter (albumin D-box) binding protein | Sec62 | NM_001034129 | SEC62 homolog (S. cerevisiae) translocation protein |
| Ddc | NM_012545 | dopa decarboxylase (aromatic L-amino acid decarboxylase) | Sep15 | NM_133297 | selenoprotein |
| Dld | NM_199385 | dihydrolipoamide dehydrogenase | Sfrs11 | NM_001035255 | serine/arginine-rich splicing factor 11 |
| Dnajc10 | NM_001106486 | DnaJ (Hsp40) homolog, subfamily C, member 10 | Slc12a2 | NM_031798 | solute carrier family 12 (sodium /potassium /chloride transporters), member 2 |
| Dstn | NM_001033666 | destrin | Spcs2 | XM_214994 | signal peptidase complex subunit 2 homolog (S. cerevisiae) |
| Eif4g1 | XM_213569 | eukaryotic translation initiation factor 4 gamma, 1 | Ssr2 | NM_001106442 | signal sequence receptor, beta |
| Epcam | NM_138541 | epithelial cell adhesion molecule | Stx1b2 | NM_012700 | syntaxin 1B |
| Erlec1 | XM_214121 | endoplasmic reticulum lectin 1 | Tm4sf12 | NM_001015026 | tetraspanin 12, transmambrane super family |
| F3 | NM_013057 | coagulation factor III (thromboplastin, tissue factor) | Tmed10 | NM_053467 | transmembrane emp24-like trafficking protein 10 (yeast) |
| Gale | NM_080783 | UDP-galactose-4-epimerase | Tmed2 | NM_031722 | transmembrane emp24 domain trafficking protein 2 |
| Gjb2 | NM_001004099 | gap junction protein, beta 2 | Tmed3 | NM_001004249 | transmembrane emp24 protein transport domain containing 3 |
| Gnpnat1 | NM_001134756 | glucosamine-phosphate N-acetyltransferase 1 | Tmed7 | XM_001063185 | transmembrane emp24 protein transport domain containing 7 |
| Insig1 | NM_022392 | insulin induced gene 1, growth response protein (CL-6) | Tmem66 | NM_001004213 | transmembrane protein 66 |
| Kcne1 | NM_012973 | potassium voltage-gated channel, Isk-related subfamily, member 1 | Tsc22d3 | NM_031345 | TSC22 domain family, member 3 |
| Kdm6b | NM_001108829 | lysine (K)-specific demethylase 6B |  |  |  |
| Laptm4b | NM_001013174 | lysosomal protein transmembrane 4 beta, lysosomal-associated protein transmembrane 4B | Tspan1 | NM_001004236 | tetraspanin 1 |
| LOC259245 | NM_147213 | alpha-2u globulin PGCL5 | Tusc3 | NM_001004212 | tumor suppressor candidate 3 |
| LOC259246 | NM_147214 | alpha-2u globulin PGCL1 | Txndc4 | NM_001008317 | thioredoxin domain containing 4 (endoplasmic reticulum) |
| LOC298111 | XM_001061142 | alpha2u globulin | Vcsa1 | NM_012684 | variable coding sequence A1, a prohormone-like protein |
| LOC689741 | XM_001071836 | similar to Der1-like domain family, member 1 | Ywhaq | NM_013053 | tyrosine 3-monooxygenase/tryptophan 5-monooxygenase activation protein, theta polypeptide |
| Lrrc41 | NM_001009710 | leucine rich repeat containing 41 | Zdhhc6 | NM_001037652 | zinc finger, DHHC domain containing 6 |
| Magt1 | NM_053946 | magnesium transporter 1 |  |  |  |
